# Exclusive Breastfeeding Practice and Associated Factors among Mothers in Boditi Town, Wolaita Zone, Southern Ethiopia, 2018: A Community-Based Cross-Sectional Study

**DOI:** 10.1155/2019/1483024

**Published:** 2019-01-01

**Authors:** Gedion Asnake Azeze, Kelemu Abebe Gelaw, Natnael Atnafu Gebeyehu, Molalegn Mesele Gesese, Taklu Marama Mokonnon

**Affiliations:** Department of Midwifery, College of Health Science, Wolaita Sodo University, Sodo, Ethiopia

## Abstract

**Background:**

Exclusive breastfeeding tops the table of life-saving interventions for newborns. A child who is exclusively breastfed is 14 times less likely to die in the first six months compared to its counterpart. Approximately 18,000 children globally still die every day and if current trend continues, some 60 million children under age 5 will die between 2017 and 2030, and half of them will be newborns. Five countries, including Ethiopia, accounted for half of all newborn deaths in the world.

**Objective:**

To assess the prevalence and associated factors of exclusive breastfeeding practice among mothers who have infants 6-12 months of age in Boditi Town, Wolaita Zone, Southern Ethiopia, 2018.

**Methods:**

Community-based cross-sectional study was conducted among 412 randomly selected mothers having 6 to 12 month infants from April 1 to 14, 2018. A pretested interviewer administered questionnaire was used for data collection. The data were entered using Epi Data version 3.1 and analyzed using SPSS version 20. Descriptive statistics was made. Bivariate and multivariate logistic regression was also carried out to see the effect of each independent variable on the dependent variable.

**Results:**

Of 412 mother-infant pairs sampled, 403 were participated, which made a response rate of 97.8%. Prevalence of EBF computed using since birth dietary recall method was 64.8% (95% C.I= 60.0, 69.0). From multivariable analysis, child birth attended by health care provider (AOR = 5.303, 95% C.I = 1.613, 17.436), postnatal care utilization (AOR = 1.91, C.I = 1.083, 3.370), and mothers who did not report any breast related problem for the first six months after child birth (AOR = 1.864, C.I = 1.090, 3.189) were factors positively associated with exclusive breastfeeding practice.

**Conclusion:**

Although the prevalence of exclusive breastfeeding practice in this study was relatively high, more effort to meet World Health Organization (WHO) recommendations is still necessary to benefit from its intervention. There is a need to promote child births to be attended by health care providers and postnatal care utilization. Further, women should be educated on what to do and where to seek care if breast problem occurs after child birth.

## 1. Background

World Health Organization (WHO) define exclusive breastfeeding (EBF) as the situation where the infant has received only breast milk for the first six months of life from his/her mother or a wet nurse or expressed breast milk and no other liquids or solids, with the exception of drops or syrups consisting of vitamins, mineral supplements, or medicines. Moreover, WHO recommends exclusive breastfeeding for 6 months, with introduction of complementary foods and continued breastfeeding thereafter [1, 2].

With the end of the era of the Millennium Development Goals (MDG) in 2015, the international community agreed on a new framework, the Sustainable Development Goal (SDG). The SDG target for child mortality represents a renewed commitment to the world's children: by 2030, end preventable deaths of newborns and children's under 5 years of age, with all countries aiming to reduce neonatal mortality to at least as low as 12 deaths per 1,000 live births and under 5 mortality to at least as low as 25 deaths per 1,000 live births. However, a child dies in every five seconds globally, approximately 18,000 children every day, and if current trend continues with more than 50 countries falling short of the SDG target on child survival, some 60 million children under age 5 will die between 2017 and 2030 and half of them will be newborns [3, 4].

Exclusive breastfeeding benefits infants by protection against infection and some chronic diseases and it leads to improved cognitive development. It is considered as unequalled way of providing ideal food for the healthy growth and development of infants. Furthermore, EBF stands out as the single most effective intervention for child survival and it tops the table of life-saving interventions for newborns. Evidence also shows that a child who is exclusively breastfed is 14 times less likely to die in the first six months compared to its counterpart [5–7]. Despite these demonstrated benefits of exclusive breastfeeding, millions of infants are not benefiting from this life-saving practice. Studies shows that the prevalence and duration of EBF in many countries [8, 9] are still lower than the international recommendations, with only 39% of infants less than 6 months of age are exclusively breastfed worldwide [10].

Globally, the largest number of newborn deaths (almost 80 percent) occurred in two regions, Southern Asia (39%) followed by sub-Saharan Africa (38%) mainly due to inadequate breastfeeding practice in combination with high level of disease. Five countries accounted for half of all newborn deaths in the world, namely India (24%), Pakistan (10%), Nigeria (9%), Democratic Republic of the Congo (4%), and Ethiopia (3%) [11].

In 2013, the Ethiopian Ministry of Health targeted to increase the proportion of exclusive breastfeeding of infants under the age of 6 months to 70% by 2015 as one strategy to improve child health [12]. However, the 2016 Ethiopian Demographic and Health Survey (EDHS) shows only 58% of mothers who had under 6 month infants exclusively breastfed [13]. So, this study was aimed to assess the prevalence and associated factors of exclusive breastfeeding practice among mothers who had infants between 6 and 12 months of age in Boditi Town, Wolaita Zone, Southern Ethiopia, 2018.

## 2. Methods and Materials

### 2.1. Study Setting and Design

Community-based cross-sectional study was employed in Boditi Town, Wolaita Zone, Southern Nations, Nationalities, and Peoples' Region (SNNPR), Ethiopia, from April 1 to 14, 2018. Boditi Town is located at 248km south of Addis Ababa (the capital city of Ethiopia) and 17km from Wolaita Soddo town. There are 9 Kebelles, smallest local administrative units, and the total population of the town in 2016, received from the local administrative office, was 53,662 (26,348 are males and 27,314 are females); from these, 18,209 were women in the reproductive age group (15-49 years). There are also 7,045 under-five age children and 2,204 infants less than one year of age. There are 3 Governmental Health center, 4 health post, 11 private clinics, 7 drug stores, 7 governmental, and 2 nongovernmental schools.

### 2.2. Study Population and Sampling Procedures

The study populations were mothers having children age between 6 and 12 months in Boditi Town during data collection period. Mothers who lived in the area at least for six months and those who gave consent to participate in the study were eligible. Mothers who are seriously ill or unable to give the required information during data collection period were excluded from the study. A single population proportion formula was used to calculate the sample size with the assumption of 58% proportion of EBF practice from 2016 EDHS [13], 95% confidence level, and 5% margin of error. After adding 10% nonresponse, the final sample size was 412.

The nine kebeles of Boditi Town comprise a total of 2603 households and from these, three kebeles; Boditi doge, Boditi Hagaza, and Fate which had 345, 275, and 185 total number of illegible infants, respectively, were selected by simple random sampling method. To select study participants from the selected three kebeles, first the sample size was proportionally allocated to size and finally a lottery method was used after taking all registered mothers who had infants of six to twelve months old from local health extension workers to select each study participant.

### 2.3. Measurements

Data were collected by face to face interview technique using a structured questionnaire. The questionnaire was prepared in English then translated to local (Amharic) language and then back to English to check for internal consistency. The questioner was constructed by adopting from previous research done on a similar topic and modified accordingly. Six preservice nursing students gathered the data and they were supervised by two Bachelor of Science (BSc.) Nursing holders. Data collectors and supervisors were oriented and trained for one day on how to interview and record the data before the start of the survey.

Since birth dietary recall method for the first six months was used for assessing exclusive breastfeeding. In this method, the participants were asked if any liquid or food other than breast milk had been given to the infant from birth to six months. In the regression analysis EBF practice was coded as “1” while “0” was coded for non-EBF practices.

### 2.4. Data Processing and Analysis

The filled questionnaires were checked for completeness and entered into Epi Data version 3.1 and transported to SPSS window version 20 for analysis. Descriptive statistics were done. Both bivariate and multivariable logistic regression models were used to identify associated factors. All variables significantly associated with exclusive breastfeeding at less than or equal to 0.2 p-values in the bivariable logistic regression model were fitted into the multivariable logistic regression model to control the effect of confounding variables. Odds ratios and their 95% CIs were computed and variables with p-value less than 0.05 were considered statistically significant.

### 2.5. Data Quality Assurance

Data quality was controlled by giving training and appropriate supervisions for data collectors. Data collectors and supervisors were local language speakers. The overall supervision was carried out by the principal investigator. A pretest was conducted on 5% of the questionnaire on one of unselected kebeles and based on a pretest result, additional adjustment was made. Appropriate modifications were made after analyzing the pretest result before the actual data collection.

### 2.6. Operational Definitions

Exclusive breastfeeding: an infant's consumption of human milk without supplementation of any water, juice, nonhuman milk, or foods except for vitamins, minerals, and medications starting from birth until six months of age.

Initiation of breast milk: the time at which breastfeeding first started.

### 2.7. Ethical Considerations

Ethical clearance was obtained from institutional review board of Wolaita Sodo University, College of Health Sciences. Official letter of permission was written to the respective study; woreda and administrative office at the selected Keble's were communicated through formal letters. Participants were informed about the purpose, benefit, risk, confidentiality of information, and the voluntary nature of participation in the study. Participants were informed that they had the right to withdraw from the study at any time and also informed verbal consent was obtained from respondents before interviewing

## 3. Result

### 3.1. Sociodemographic Characteristics of Participants

Of 412 mother-infant pairs sampled, 403 were participated in this study, which made a response rate of 97.8%. The mean age of mothers in the study is found to be 28.02 years (28.02±5.89 standard deviation [SD]). Most of the study participants 360(89.3%) were residing in urban area while the remaining was from rural goxs of urban kebeles. Of the study participants, 225 (55.8%) were married and 210 (52.1%) found in occupational group of house wife. Concerning ethnicity, among the participants, 252 (62.5%) were Wolaita/SNNPR followed by Gurage 49 (12.2%), Oromo 60 (14.9%), and Amhara 35 (8.7 %) and the rest 7 (1.7%) were others. The average household monthly income of the respondents was 2244.91 Ethiopian Birr per month (standard deviation [SD] ±1841.59), and 207 (51.1%) respondents earn above 1000 Ethiopian Birr per month. Of the infants studied, the age distributions of female and male infants were more or less equal, 209 (51.9%) females and 194 (48.1%) males. The sociodemographic characteristics of the study population are listed in Table 1.

### 3.2. Maternal-Infant Related Characteristics of Participants

From the study participants, three hundred nineteen (79.2%) reported their HIV status as negative while the remaining 21 (5.5%) and 63 (15.6%) reported their HIV status as positive and unknown status, respectively. Of the study participants, about 80.6% of mothers had made at least one Antenal Care (ANC) visit during pregnancy and from these, nearly three-quarter (72%) of them received breastfeeding counseling. One-third (35.5%) of mothers did not have any postnatal checkup after delivery. Nevertheless, of the mothers who received postnatal care (260/64.5%), around two hundred twenty-five (55.8%) of them were told about infant feeding up to six months of age. Majority (81.1%) of mothers were attended by health care provider during the time of child birth (Table 2).

### 3.3. Breastfeeding and Related Characteristics of the Participant

Almost all (95.3%) of study participants responded that they heard about EBF at some point in time and half (49.9%) of them mentioned community health workers as their primary source of information. Furthermore, finding of this study shows that, of the participants who had ever breastfed (96.8%) the current child, almost half (47.1%) of them put their child to the breast in the first one hour of life but thirteen mothers had never put their infant to their breast. In addition, when study participant asked about whether they had a plan during pregnancy to exclusively breastfeed, around three-quarter (76.7%) of mothers had a plan to breastfeed exclusively and about one hundred ninety-eight (49.1%) did not have breastfeeding history before. A total of two hundred eighty-three (70.2%) and majority (86.1%) of mothers reported they had their husband and cultural/religious support with regard to breastfeeding their infant exclusively and around two-third (66.3%) of study participants do not have a history of contraception use within the first six months of child birth (Table 3).

The prevalence of EBF computed using since birth dietary recall method showed two hundred sixty-one (64.8% with 95% C.I 60.0, 69.0) of the participants practiced EBF.

### 3.4. Factors Associated with Exclusive Breastfeeding Practice

#### 3.4.1. Multivariable Analysis

In this section the effects of factors that have suspected relationship to exclusive breastfeeding practice have been explored using both bivariate and multivariable analysis. After identifying the nominee predictor variables of EBF practice of mothers using bivariate logistic regression statistical test, we developed a model to control the possible confounder variables. Accordingly variables which made an association in bivariate analysis with p-value <0.2 were residence, mothers educational status, total number of under 5 children, ANC utilization, number of ANC visits, place of delivery, birth attendant, PNC utilization, ever heard about EBF, how soon after birth did the mother put newborn to breastfeed, colostrum feeding, decision maker on infant feeding, breast problem in the first six months of birth, contraception use in the first six months of birth, husband support, and cultural/religious support (Table 4).

Those significant factors found during bivariate analysis were considered together in multivariable analysis. Upon fitting the factors using binary logistic regression, three (3) factors made significant association with EBF practice in the final analysis with p-value <0.05. These include birth attendants, PNC utilization, and breast problem in the first six months of birth (Table 4).

The odds of EBF practice were higher among those women attended by health care provider during the birth of the indexed infant, where the odds of practicing EBF were more than fivefold among mothers attended by health care provider (AOR = 5.27, 95% C.I = 1.618, 17.211), than those who were attended by traditional birth attendants/relatives.

Furthermore the finding of this study also shows that mothers who had reported PNC service utilization were 82% more likely to practice EBF (AOR = 1.82, C.I = 1.049, 3.163) as compared to those mothers who did not received. Moreover, the odds of mothers who do not report breast problem in their first six months of child birth were 86% more likely (AOR = 1.86, C.I = 1.096, 3.187) to practice EBF than those who had reported.

## 4. Discussion

### 4.1. Exclusive Breastfeeding Practice

In this study, the overall prevalence of exclusive breastfeeding was 261 (64.8%) (95% CI of 60.0, 69.0). The prevalence was lower than studies conducted in Ethiopia; Goba district 71.3% [14], Debre Tabor town 70.8% [15], and Ambo Woreda Oromia Region 82.2% [10]. The observed variation might be due to methodological, time, or cultural differences across areas and between studies. For instance, from the study conducted at Goba district Ethiopia [14], the authors measured prevalence of EBF by taking history of infant feeding for the last 24 hours whereas in this study, mothers were requested by the data collectors on their practice on dietary recall for the first six month of life of their index baby aged 6 to 12 months. Hence, the point estimate might be inflated on the study conducted at Goba district whereas the recall since birth method used to determine EBF in this study might have underestimated the EBF prevalence. Furthermore, studies shows that a single 24-hour recall tends to overestimate the EBF rates [16, 17]. This explanation can be further strengthened by another study where the percentage of women who reported to have EBF their infants over the previous 24h were 70.2% while the same study shows percentage of EBF since birth were 51.6% [18].

The finding of this study is congruent with study finding level of 68.6% in Debre Berhan District, Central Ethiopia [19], 64% in Gozamen District [20], 60.8% in Debre Markos town [21], 63.2% of a study conducted in Juba, South Sudan [22], and 61.5% of Urban Slum in Western India [23]. The observed similarity between these findings might be due to the fact that most of the studies were conducted in the same country and continent and it is believed that these countries will not have significant difference in their sociodemographic characteristics.

This finding was higher as compared with study conducted in Ethiopia; 58% of the 2016 National prevalence of EDHS [13], 50.3% of Bahir Dar City, Northwest Ethiopia [5], and 44.2% of Addis Ababa [1]. This difference could be the result of efforts and multisectoral collaborations that have been made by the Government of Ethiopia on child nutrition since 2013 while the study mentioned above [5] used data which was collected before the implementation of these revised national nutrition programs. Methodological difference might be another reason for this observed variation in findings. For instance, an institutional-based cross-sectional study design was used in a study conducted at Addis Ababa, Ethiopia [1]. As well, the results of this study were also higher when compared with findings conducted in China 44.9% [24], Tehran, Islamic Republic of Iran, 46.5% [25], Democratic Republic of the Congo 49.2% [26], Accra Ghana, 51.6% [18], and Kumasi Metropolis of Ghana, 48% [27]. The difference might be due to methodological variations between studies and differences in sociocultural, economical, health, and health service utilization characteristics between respondents of the referenced areas and the study place. For instance, on the above studies [24, 26] women having less than 5 years old child were selected to assess EBF for the first six months of infant's life in which the result might be prone to recall bias.

### 4.2. Factors Associated with Exclusive Breastfeeding Practice

The key factors that were statistically associated with higher rates of EBF included birth attendants, PNC utilization, and breast problem in the first six months of birth.

The finding of this study indicates that the odds of practicing EBF among those women who were attended by health care provider during child birth were 5 times (AOR = 5.27, 95% C.I = 1.618, 17.211) than those who were attended by traditional birth attendants/relatives. To the best of our knowledge, there was no other similar study finding which shows the association between child birth attendant and EBF practice. However, our finding differed with findings from Bangladesh where deliveries assisted by relatives had lower risk of terminating breastfeeding than by the health professionals [28]. One possible explanation for the observed association in our study could be that mothers who gave birth by the help of health care provider might get the opportunity to be counseled by the delivery attendant on the benefits of early initiation and maintenance of breastfeeding, colostrum feeding, proper positioning, and attachment. This in turn may increase the practice of EBF, as evidenced in other studies [29, 30]. Another explanation may be due to the fact that the risk of birth trauma and postpartum complications may also get reduced when mothers receive assistance from healthcare provider during childbirth which has the potential to influence breastfeeding practice.

Postnatal care utilization was found to be predictor of EBF practice where those who had reported PNC follow-up were 90% more likely (AOR = 1.91, C.I = 1.083, 3.370) to breastfeed exclusively for the first six months of life as compared to those mothers who did not received PNC. This finding is in line with study conducted in Halaba Special woreda, SNNPR, Ethiopia (AOR=2.2; 95%C.I=1.25, 3.87) [31]. This might be due to the assumption that the 2013 WHO recommendations on postnatal care suggest mothers should be counseled and provided support for exclusive breastfeeding at each postnatal contact [30] and if they could have got the chance, counseling during PNC visits may enhances mothers' understanding and appreciation of the demands and benefits of EBF.

Moreover, those respondents who did not report breast problem in their first six months of child birth were 86% more likely (AOR = 1.864, C.I = 1.090, 3.189) to practice EBF than those who had reported breast problem for the indicated period. This finding was supported with a prospective cohort study conducted in central Nepal (Hazard Ratio, (HR)=2.07; 95% C.I=1.66, 2.57) [32]. This might be due to the fact that when women had breast related problems, they may consider that the quality of milk was affected or it was insufficient, a reason for most mothers to prematurely cease breastfeeding and introducing other foods before six months.

## 5. Conclusion and Recommendation

Although the prevalence of exclusive breastfeeding practice in this study was relatively high, more effort is still necessary to meet the Ethiopian Health Sector Development Program (HSDP) IV target level of 70% and WHO recommendations of 90%. Birth attendant, PNC utilization, and breast related problems for the first six months after child birth were independent predictors of exclusive breastfeeding practice.

We suggest health institutions and health care providers to encourage and promote PNC utilization. Child births should be attended by health care personnel in order to intensify efforts at informing women about the importance of breastfeeding, especially EBF. Health education during pregnancy and immediately after delivery on developing a health seeking behavior among women who develop breast problems after child birth is important. Further, women should be educated on what to do and where to seek care if breast problem arises.

## Figures and Tables

**Table 1:** Sociodemographic characteristics among mothers who had 6-12 months infants in Wolaita Zone, Boditi Town, Southern Ethiopia, 2018.

VARIABLES N=403	FREQUENCY (n)	PERCENTAGE (%)
**Age of the mother **		
≤24	103	25.6
25-29	141	35.0
30-34	109	27.0
≥35	50	12.4
**Marital status**		
Married	225	55.8
Married but separated	55	13.6
Single	55	13.6
Cohabiting	23	5.7
Divorced	30	7.4
Widowed	15	3.7
**Religion **		
Protestant	236	58.6
Orthodox Christian	95	23.6
Muslim	36	8.9
Catholic	23	5.7
Others, specify^1^	13	3.2
**Mother's occupational status**		
Housewife	227	56.3
Merchant	107	26.6
Government employee	33	8.2
Privet organization	26	6.5
Others, specify^2^	10	2.5
**Father's occupational status**		
Merchant	214	53.1
Farmer	45	11.2
Government employee	79	19.6
Privet employee	56	13.9
Others, specify^3^	9	2.2
**Mother's Educational status **		
Unable to read and write	77	19.1
Read and write	33	8.2
Primary education	128	31.8
Secondary education	99	24.6
Tertiary education and above	66	16.4
**Husband's educational status **		
Unable to read and write	38	9.4
Read and write	33	8.7
Primary education	93	23.1
Secondary education	98	24.3
Tertiary education and above	139	34.5
**Household average monthly Income **		
≤1000	135	33.5
1001-2000	125	31.0
≥2001	143	35.5

^1^ 7^th^ day Adventist and traditional. ^2^Not working, daily worker, and student. ^3^Daily worker, tailor, and student.

**Table 2:** Maternal-infant related characteristics among mothers who had 6-12 months infants in Wolaita Zone, Boditi Town, Southern Ethiopia, 2018.

VARIABLES N=403	FREQUENCY (n)	PERCENTAGE (%)
**Total number of under 5 children**		
One	236	58.6
Two	123	30.5
Three and above	44	10.9
**Birth order of this infant**		
First	188	46.7
Second	86	21.3
Third	67	16.6
Fourth and above	62	15.4
**Birth interval from the preceding birth**		
First Pregnancy	188	46.7
≤3 years	167	41.4
>3 years	48	11.9
**ANC service utilization**		
No	78	19.4
Yes	325	80.6
**Number of ANC visits**		
Never Had	78	19.4
Once	26	6.5
Two times	85	21.1
Three times	81	20.1
Four times and above	133	33.0
**Counseling on infant feeding during ANC visit **		
No	91	28
Yes	234	72
**Place of delivery**		
Home	104	25.8
Health Institutions	299	74.2
**Mode of delivery**		
Normal/Vaginal	339	84.1
Cesarean section	64	15.9
**Counseling regarding infant feeding during PNC**		
No	35	8.7
Yes	225	55.8

ANC: antenatal care.   PNC: postnatal care.

**Table 3:** Breastfeeding and related characteristics among mothers who had 6-12 months infants in Wolaita Zone, Boditi Town, Southern Ethiopia, 2018.

VARIABLES N=403	FREQUENCY (n)	PERCENTAGE (%)
**Ever heard about EBF**		
No	19	4.7
Yes	384	95.3
**Primary source of information** **∗**		
Community Health Worker/Health worker	201	49.9
Mass Media	86	21.3
Husband/Family	19	4.7
Neighbors/Friends	26	6.5
Health Development Army	42	10.4
School	10	2.5
**Exclusive breastfeeding plan during pregnancy**		
No	94	23.3
Yes	309	76.7
**Previous experience on breastfeeding**		
No	198	49.1
Yes	205	50.9
**Starting breastfeeding at hospital/health center** *∗*		
No	61	20.4
Yes	238	79.6
**Breast problem within the first 6 months of birth**		
No	273	67.7
Yes	130	32.3
**How soon after birth did you put your infant to breast**		
Not breastfeed ever	13	3.2
Within one hour of birth	190	47.1
1 hour up to 1 day	132	32.8
1 day up to 3 days	68	16.9
**Contraception use within the first six months of child birth**		
Yes	136	33.7
No	267	66.3
**Colostrum feeding **		
No	111	27.5
Yes	292	72.5
**Husband support for EBF**		
No	120	29.8
Yes	283	70.2
**Cultural/religious support for EBF**		
No	56	13.9
Yes	347	86.1
**Decision maker on infant feeding **		
My own decision	206	51.1
Husband	70	17.4
Together with husband	60	14.9
Health care provider	48	11.9
Others, specify^2^	19	4.7

HC: health center. *∗*Respondents were asked if they responded “yes” to the previous questions in the questionnaire. ^2^Relatives, friends, and religious fathers.

**Table 4:** Bivariate and multivariable logistic regression results on factors associated with exclusive breastfeeding practice among mothers who had 6-12 months infants in Wolaita Zone, Boditi Town, Southern Ethiopia, 2018.

Variables	**Exclusive Breastfeeding **		Crude OR	Adjusted OR
	**No (**%**)**	**Yes (**%**)**	(95% C.I)	(95% C.I)
**Residence **				
Urban	122(33.9)	238(66.1)	1	1
Rural	20(46.5)	23(53.5)	0.58(0.312, 1.115)	1.07(0.457, 2.550)
**Mothers education status**				
Unable to read and write	35(45.5)	42(54.5)	1	1
Read and write	12(36.4)	21(63.6)	1.45(0.630, 3.375)	1.11(0.415, 3.324)
Primary education	39(30.5)	89(69.5)	1.90(1.059, 3.415)	1.23(0.569, 2.668)
Secondary education	33(33.3)	66(66.7)	1.66(0.903, 3.077)	0.87(0.377, 2.011)
Tertiary and above	23(34.8)	43(65.2)	1.55(0.792, 3.064)	0.98(0.408, 2.381)
**Total Under 5 children **				
One	73(30.9)	163(69.1)	1.69(0.879, 3.274)	0.83(0.373, 1.872)
Two	50(40.7)	73(59.3)	1.11(0.553, 2.227)	0.46(0.194, 1.099)
Three and above	19(43.2)	25(56.8)	1	1
**ANC service utilization**				
No	35(44.9)	43(55.1)	1	1
Yes	107(32.9)	218(67.1)	1.65(1.003, 2.741)	0.99 (0.459, 2.155)
**No of ANC visits**				
Never had	35(44.9)	43(55.1)	1	1
Once	15(57.7)	11(42.3)	0.59(0.243, 1.464)	0.48(0.163, 1.423)
Twice	33(38.8)	52(61.2)	1.28(0.687, 2.394)	0.73(0.362, 1.501)
Three times	27(33.3)	54(66.7)	1.62(0.856, 3.094)	1.01(0.500, 2.070)
Four times and above	32(24.1)	101(75.9)	2.56(1.413, 4.670)	1.52(0231, 4.658)
**Place of birth **				
Home	51(49.0)	53(51.0)	1	1
Health Institution	91(30.4)	208(69.6)	2.19(1.393, 3.472)	0.44(0.151, 1.313)
**Birth attendant **				
Traditional birth attendant	44(57.9)	32(42.1)	1	1
Health care professional	98(30.0)	229(70.0)	3.21(1.923, 5.367)	5.27(1.618,17.211) *∗∗*
**PNC Utilization**				
No	69(48.3)	74(51.7)	1	1
Yes	73(28.1)	187(71.9)	2.38(1.561, 3.654)	1.82(1.049, 3.163)*∗*
**Ever heard about EBF**				
No	13(68.4)	6(31.6)	1	1
Yes	129(33.6)	225(66.4)	4.28(1.591,11.53)	1.59(0.473, 5.394)
**How soon after birth did you put your infant to breast **				
Never put	7(53.8)	6(46.2)	1	1
Within one hour of birth	35(18.4)	155(81.6)	5.16 (1.635,16.325)	2.09 (0.546, 8.043)
1 hour up to 1 day	57(43.2)	75(56.8)	1.53(0.489, 4.817)	0.73(0.190, 2.810)
1 day up to 3 days	43(63.2)	25(36.8)	0.67(0.205, 2.245)	0.36(0.087, 1.495)
**Colostrum feeding**				
No	60(54.1)	51(45.9)	1	1
Yes	82(28.1)	210(71.9)	3.01(1.917, 4.736)	1.48(0.811, 2.732)
**Decision maker on infant feed**				
My own decision	52(26.5)	144(73.5)	2.90(1.122, 7.517)	1.35(0.434, 4.238)
Husband/spouse	23(34.8)	43(65.2)	2.00(0.718, 5.572)	1.03(0.303, 3.554)
Together with husband	33(55.0)	27(45.0)	0.90(0.323, 2.557)	0.61(0.175, 2.135)
Health care provider	17(35.4)	31(64.6)	2.02(0.690, 5.951)	1.02(0.284, 3.676)
Others, specify^1^	17(51.5)	16(48.5)	1	1
**Breast problem since birth**				
Yes	67(51.5)	63(48.5)	1	1
No	75(27.5)	198(72.5)	2.80(1.818, 4.336)	1.86(1.096, 3.187)*∗*
**Contraception use within the first 6 months of birth**				
Yes	61(44.9)	75(55.1)	1	1
No	81(30.3)	186(69.7)	1.86(1.219, 2.862)	1.68(0.984, 2.869)
**Husband support for EBF**				
No	64(53.3)	56(46.7)	1	1
Yes	78(27.6)	205(72.4)	3.00(1.928, 4.680)	1.05 (0.582, 1.927)
**Cultural/religious support**				
No	31(55.4)	25(44.6)	1	1
Yes	111(32.0)	236(68.0)	2.63(1.486, 4.676)	1.71(0.789, 3.712)

^*∗*^Significant association at P-value <0.05; *∗∗*strong association with Adjusted Odds Ratio ≥3.

^1^Relatives, friends, and religious fathers.

## Data Availability

All data are already described and included in the manuscript.

## Abbreviations

ANC: Antenatal Care
AOR: Adjusted Odd Ratio
CI: Confidence Interval
COR: Crudes Odds Ratio
EBF: Exclusive breastfeeding
EDHS: Ethiopian Demographic and Health Survey
NGO: Nongovernmental Organization
PNC: Postnatal care
SDG: Sustainable Development Goal
SNNPR: Southern Nations, Nationalities, and Peoples' Region
UNICEF: United Nations International Children's Emergency Fund
WHO: World Health Organization.

## Additional Points

*Limitations. *A limitation of this study was that it assessed only the quantitative aspects of exclusive breastfeeding and it may also have underestimated, or even may overestimate, the prevalence of exclusive breastfeeding in the study area due to recall bias. Data on EBF also relied on self-report by the women and therefore, reporting bias may have also influenced the results.
